# A study on social determinants of infant mortality in Malaysia

**DOI:** 10.1186/1471-2458-12-S2-A33

**Published:** 2012-11-27

**Authors:** Amaluddin Ahmad

**Affiliations:** 1 School of Medical Sciences, Cyberjaya University College of Medical Sciences, 63000 Cyberjaya, Malaysia

## Background

There is a large body of empirical evidence to suggest that social conditions are one of the major determinants of population health. This study aimed to examine the relationship between social determinants and infant mortality in Malaysia.

## Materials and methods

Statistical analysis of infant mortality and social determinants data using correlations, factor analysis and multiple regressions were undertaken in order to examine the collective influence of a range of social determinants indices on variations observed in infant mortality. Determinants of infant mortality in Malaysia tested in this study include GDP per capita, poverty rate, mean income of bottom 40% income earner, Gini coefficient, ratio of top 20% income: to bottom 40% income, population per doctor ratio, hospital bed per population ratio, car ownership per population, computer ownership per population, urbanization rate, percentage living in single housing and flats, women education and social development index.

## Results

Simple regression revealed significant relation between IMR with fifteen predictors but multiple regressions failed to demonstrate any significant linear relationship because of the multicollinearity. Factor analysis was done and new variables were created based on the identified factors. With the new group of variables, economic development explained 27%, socioeconomic status explained 21%, income inequality explained 14%, service provision 9% and finally type of housing explained 4% of the variability observed in IMR. However, collectively, the variables were able to explain only 29% of the variability in IMR using multiple linear regressions analysis.

## Conclusions

Developing a better understanding of the social determinants of health is critical in order to ameliorate the social determinants associated with poor health and to reduce the health disparities within the population.

